# Modelling Survival and Mortality Risk to 15 Years of Age for a National Cohort of Children with Serious Congenital Heart Defects Diagnosed in Infancy

**DOI:** 10.1371/journal.pone.0106806

**Published:** 2014-09-10

**Authors:** Rachel L. Knowles, Catherine Bull, Christopher Wren, Angela Wade, Harvey Goldstein, Carol Dezateux, on behalf of the UKCSCHD (UK Collaborative Study of Congenital Heart Defects) collaborators

**Affiliations:** 1 Population Policy and Practice Programme, Institute of Child Health, University College London, London, United Kingdom; 2 Cardiac Unit, Great Ormond Street Hospital for Children NHS Trust, London, United Kingdom; 3 Department of Paediatric Cardiology, Freeman Hospital, Newcastle-upon-Tyne, United Kingdom; Piazza S, Italy

## Abstract

**Background:**

Congenital heart defects (CHDs) are a significant cause of death in infancy. Although contemporary management ensures that 80% of affected children reach adulthood, post-infant mortality and factors associated with death during childhood are not well-characterised. Using data from a UK-wide multicentre birth cohort of children with serious CHDs, we observed survival and investigated independent predictors of mortality up to age 15 years.

**Methods:**

Data were extracted retrospectively from hospital records and death certificates of 3,897 children (57% boys) in a prospectively identified cohort, born 1992–1995 with CHDs requiring intervention or resulting in death before age one year. A discrete-time survival model accounted for time-varying predictors; hazards ratios were estimated for mortality. Incomplete data were addressed through multilevel multiple imputation.

**Findings:**

By age 15 years, 932 children had died; 144 died without any procedure. Survival to one year was 79.8% (95% confidence intervals [CI] 78.5, 81.1%) and to 15 years was 71.7% (63.9, 73.4%), with variation by cardiac diagnosis. Importantly, 20% of cohort deaths occurred after age one year. Models using imputed data (including all children from birth) demonstrated higher mortality risk as independently associated with cardiac diagnosis, female sex, preterm birth, having additional cardiac defects or non-cardiac malformations. In models excluding children who had no procedure, additional predictors of higher mortality were younger age at first procedure, lower weight or height, longer cardiopulmonary bypass or circulatory arrest duration, and peri-procedural complications; non-cardiac malformations were no longer significant.

**Interpretation:**

We confirm the high mortality risk associated with CHDs in the first year of life and demonstrate an important persisting risk of death throughout childhood. Late mortality may be underestimated by procedure-based audit focusing on shorter-term surgical outcomes. National monitoring systems should emphasise the importance of routinely capturing longer-term survival and exploring the mechanisms of mortality risk in children with serious CHDs.

## Introduction

Serious congenital heart defects (CHDs), requiring surgery in the first year of life, affect around 1% of births each year in the UK [1]–[3] and include a broad spectrum of complexity and severity. Without intervention in early life, serious CHDs are often incompatible with long-term survival. Continuous improvement in medical, intensive care and surgical technologies have significantly reduced infant mortality and around 80% of affected babies now survive the first year of life [4]–[7]. Despite the rising number of paediatric cardiac procedures being undertaken [8], surgical mortality is decreasing and the number of UK adults with CHDs is steadily growing [9]. Nevertheless, long-term survival for individuals within most CHD subgroups remains below that of the unaffected population [10].

Recognising the growing population of adolescents and adults with CHDs, recent research has focused on the epidemiology and health experience of adult survivors. It is generally assumed that additional mortality between infancy and 15 years of age is extremely low and likely to be related to further surgery, yet observational studies with prospective long-term follow-up of the transition of children with CHDs from infancy through school, adolescence and into adulthood are lacking. While short-term surgical outcome studies provide extensive detail about post-operative survival and mortality, particularly within specific CHD subgroups, the factors associated with mortality risk that are common to all diagnostic subgroups and their significance at different ages are poorly defined [11]. Developing a life course approach [12] to children and adults surviving with CHDs is a vital step in recognising that they experience complex interactions between intrinsic early life factors, cardiac diagnosis and the pressures of the external environment, which have a significant impact on their growth, development and late outcomes. There remains a crucial lack in our understanding of the relevance of different risk factors and exposures at each stage of the lifecourse and how these may modify long-term outcomes, not only when they occur in relation to critical periods but also when experienced as repeated exposures such as multiple surgical interventions. Such information is key to future interventions to improve survival and health outcomes and ensure a smooth transition to adult life for affected children.

The UK Collaborative Study of Congenital Heart Defects (UKCSCHD) was established as a UK-wide multi-centre cohort involving almost 4000 children, diagnosed with serious CHDs during their first year of life, to determine prospectively survival, health, educational and quality of life outcomes. In this paper we examine survival from birth to 15 years of age, characterise the timing and causes of death during childhood and investigate the relative importance of patient-specific and early life factors to mortality risk during childhood for this national cohort that is largely representative of contemporary surgical and medical management.

## Materials and Methods

### Ethics statement

Ethics approval was granted by Trent Multicentre Research Ethics Committee (MREC 04/4/017). Case notes review was undertaken within collaborating centres under the supervision of the local responsible cardiologist; the names and addresses of the children were not provided to the central study team who carried out analyses on de-identified data.

### Methods

The UKCSCHD includes children with serious CHDs born between 1992 and 1995 and prospectively notified to a UK-wide study evaluating fetal diagnosis by paediatric cardiologists (British Congenital Cardiac Association [BCCA] members) in all 17 UK paediatric cardiac surgical centres [13]. Serious CHDs were defined as structural heart malformations requiring intervention or resulting in death during the first year of life [13]

A retrospective hospital records review of 3897 children was co-ordinated by the central research team; 268 children from the original register were excluded as there were insufficient details in the case notes to confirm that all inclusion criteria (Table 1) for the UKCSCHD were met. Local clinicians extracted data and completed a standardised proforma for 3698 (95%) of 3897 children. Record retrieval varied by centre, reflecting local record-keeping, and retrieval was less successful for children who were reported by local clinicians to have died (difference 6% [95% CI 4%, 8%]).

**Table 1:** Inclusion and exclusion criteria for the UKCSCHD cohort.

Inclusion criteria (for the UKCSCHD and original register). **Live born infants:**
1) born between 1^st^ January 1992 and 31^st^ December 1995
2) resident in the United Kingdom (UK) at birth
3) with a serious congenital heart defect, defined as a structural malformation of the heart or great vessels, requiring an intervention or resulting in death during the first year of life.

Exclusion criteria (for the UKCSCHD and original register). **Live born infants with:**
1) isolated cardiovascular defects such as persistent ductus arteriosus, vascular ring or anomalous coronary arteries
2) myocardial dysfunction, arrhythmia or tumours in a structurally normal heart
3) a congenital heart defect that did not require an intervention or result in death during the first year of life.

*Excluded from the UKCSCHD but included in the original register:*
*Babies with a fetal diagnosis of serious congenital heart defect made at prenatal ultrasound but not subsequently live born.*

Deaths were traced within hospital systems then validated through the Office for National Statistics (England and Wales) and General Register Office (Scotland). A primary cardiac diagnosis (Table 2) was assigned to every child using a hierarchical classification prioritising the most severe structural defect adapted from Wren et al. [14]; three clinical raters (CB, CW, RK) independently assigned diagnoses (generalised kappa (κ) for rater agreement = 0.83) and the final diagnosis was agreed by consensus. Two cardiologists (CB, CW) designated one procedure for each child as ‘definitive’, defined as the procedure which would approximate normal anatomy and restore biventricular function or provide long-term palliation without expectation of further surgery during childhood, thus the final stage of a multi-stage repair was considered definitive. The definitive procedure may have taken place at any time during follow-up. Children were assigned to a cardiac prognostic severity (CPS) group, adapted from Lane [15], based on primary diagnosis and whether their definitive procedure was presumed curative, corrective or palliative (Table 2).

**Table 2:** Cardiac diagnosis and severity for all children in the cohort.

PRIMARY CHD DIAGNOSIS*		Number of children	*Cardiac Prognostic Severity Group* **				
			**(% of children within diagnostic group)**				
			***Curative***	***Corrective***	***Palliative***	***No intervention***	***Insufficient information***
HLH/MA	Hypoplastic left heart/mitral atresia	199	*0*	*0*	*65%*	*32%*	*4%*
TA	Tricuspid atresia	67	*0*	*0*	*97%*	*3%*	*0*
DIV	Double inlet ventricle	85	*0*	*0*	*96%*	*4%*	*0*
PA+IVS	Pulmonary atresia with intact ventricular septum	83	*0*	*0*	*99%*	*1%*	*0*
PA+VSD	Pulmonary atresia with ventricular septal defect	151	*0*	*34%*	*62%*	*3%*	*1%*
CAT	Common arterial trunk (Truncus arteriosus)	99	*0*	*89%*	*6%*	*5%*	*0*
CAVSD	Complete atrioventricular septal defect	460	*0*	*74%*	*21%*	*5%*	*0*
TGA	Transposition of the great arteries	597	*0*	*83%*	*17%*	*0%*	*0*
TOF	Tetralogy of Fallot	361	*0*	*81%*	*17%*	*1%*	*1%*
TAPVC	Total anomalous pulmonary venous connection	150	*93%*	*4%*	*1%*	*1%*	*0*
VSD	Ventricular septal defect	760	*63%*	*25%*	*11%*	*2%*	*0*
AS	Aortic stenosis	107	*0*	*92%*	*7%*	*2%*	*0*
PS	Pulmonary stenosis	194	*95%*	*2%*	*2%*	*1%*	*0*
COA	Coarctation of the aorta	395	*0*	*97%*	*1%*	*1%*	*0*
Misc	Miscellaneous	189	*21%*	*25%*	*46%*	*8%*	*1%*
	**Total**	**3,897**					

**Notes**:

*adapted from Wren [14];

**adapted from Lane [15].

**Assignment of primary CHD diagnosis**: The methodology for assigning primary diagnoses to 1,768 children with multiple defects was validated independently by three raters (RK, CB, CW). Based on cardiac diagnoses in medical records, children assigned a primary diagnosis were 1,738 (98%), 1,610 (91%) and 1,146 (65%) for each rater; this increased to 1,761 (99.7%), 1,689 (95.5%) and 1,658 (93.8%) respectively using records of surgical procedures (Interrater agreement: k = 0.83). A ‘miscellaneous’ category included defects found in fewer than 40 children: congenitally corrected transposition of the great arteries (n = 24), partial atrioventricular septal defect (n = 20), aortopulmonary window (n = 26), atrial septal defect (n = 36) and rarer diagnoses (n = 83).

**CPS groups were**: *no intervention-*children who received no surgical intervention prior to death during first year of life; *curative*-children who had successful repair of atrial or ventricular septal defect, pulmonary stenosis or total anomalous pulmonary veins and had no additional cardiac defects; *corrective-*children who had a procedure which approximated normal anatomy and restored biventricular function, with no expectation of future surgery during childhood; *palliative-*children whose surgery did not restore biventricular function, including children for whom all stages of multi-stage repair were not achieved, who had a valve replacement which would require later revision, or for whom only a single functional ventricle circulation was possible.

### Statistical analysis

Descriptive statistics are presented as numbers and percentages, or median and interquartile ranges (IQR), and 95% confidence intervals (CI) were estimated for the difference between two proportions. Table 3 provides information about the numbers of children who died or were censored alive (last seen) during each year of follow-up. The Kaplan-Meier survival function was estimated up to 15 years of age; five children, whose date of death was not known, were censored alive on the date of the last hospital visit.

**Table 3:** Number of children dying or last seen (censored) during each year of follow-up.

Year of follow-up	Number at risk	Deaths	Censored (last seen)
*(from birth = 0)*	*n*	*n*	*n*
0–1 year	3897	727	681
1–2 years	2489	78	103
2–3 years	2308	35	55
3–4 years	2218	28	46
4–5 years	2144	16	58
5–6 years	2070	11	53
6–7 years	2006	8	48
7–8 years	1950	8	64
8–9 years	1878	3	106
9–10 years	1769	5	168
10–11 years	1596	4	405
11–12 years	1187	4	520
12–13 years*	663	4	424
13–14 years*	235	1	214
14–15 years*	20	0	20

*All children in the cohort were aged 12 years or older at the time of ascertainment of deaths in 2007, thus losses from follow-up at younger ages were due to death or censoring alive on the date last seen (as recorded in hospital case notes). Between 12 and 15 years there are fewer children under follow-up (‘at risk’) in the older age groups as many children had not reached these ages and this reduced the precision of survival estimates after 12 years.

A multilevel discrete time event history model was developed to investigate the factors influencing survival; this was a binomial logit model with the response variable, death or censoring, coded as a binary variable. The discrete time hazard function represented the conditional probability of an event in each interval given that the event had not occurred in a previous interval: *h*(*t*)  = Pr(*T* = *t* | *T*≥*t*). The period of observation was divided into 24 discrete intervals; 12 intervals of one month duration for the first year of life when the majority of events occurred, then 11 intervals of one year from one to 12 years of age and a final interval of three years duration representing the interval 12 to 15 years of age, in which events were rare. In the final model including multiple predictors, three ‘smoothed’ categories (representing the first year of life, one to 12 years, and 12 to 15 years of age) were developed by estimating the log-transformed midpoint of each variable and dividing by the number of months within each category. Although the 24 category model had marginally better fit than the ‘smoothed’ category model (DIC 8887.9 and 8946.8 respectively), the smoothed category model improved model convergence and was therefore used.

Within each interval, it was assumed that the hazards were constant and the binary response variable indicated whether death occurred during the final interval. Covariates specific to the child, such as sex or preterm birth, remained constant whilst factors recorded at each procedure, such as cardiopulmonary bypass duration, were permitted to vary between intervals. Weight and height were converted to age- and sex-standardised z-scores (British 1990 growth reference) [16]. Each predictor was explored in univariable analyses then multivariable models were constructed to determine joint associations.

Missing data were imputed using a hierarchical imputation model developed with MLwiN 2.18–2.20 [17] and Realcom-Impute [18]. The imputation procedure used Markov-chain Monte Carlo (MCMC) procedure to generate 20 imputed datasets during 2500 iterations. Imputation was conditioned on centre availability of records, as this significantly influenced missingness. Imputation excluded 28 children whose date of death or censoring was unknown, and variables with more than 50% missing data; the included variable with the most missing data was clinical status on admission (46%; Table 4). Distributions of continuous variables before and after imputation were compared using density plots (data not shown).

**Table 4:** Patient-specific characteristics by diagnostic group (n = 3897).

Primary CHD Diagnosis	Boys	Preterm	DS: Down's syndrome	Non-DS non-cardiac malformation	Add. cardiac defects	Unstable clinical status	Age at first intervention*	Death without intervention
	*as % of all non-missing values* †						Median (IQR) days	n
**HLH/MA**	67%	7%	0	4%	26%	75%	5 (2,14)	63
**TA**	52%	6%	0	6%	88%	57%	23 (5,83)	2
**DIV**	71%	4%	0	7%	92%	64%	14 (5,46)	3
**PA+IVS**	64%	8%	0	5%	29%	82%	3 (2,6)	1
**PA+VSD**	45%	10%	0	18%	52%	61%	8 (3,81)	5
**CAT**	52%	10%	0	17%	40%	61%	29 (12,65)	5
**CAVSD**	47%	8%	43%	5%	43%	42%	103 (55,169)	22
**TGA**	68%	5%	0	2%	51%	65%	3 (1,14)	1
**TOF**	59%	10%	3%	12%	29%	33%	133 (42,249)	4
**TAPVC**	64%	7%	0	6%	19%	76%	16 (3,62)	2
**VSD**	51%	9%	9%	10%	54%	57%	97 (34,180)	13
**AS**	70%	2%	2%	5%	44%	60%	15 (3,61)	2
**PS**	45%	6%	1%	8%	27%	23%	72 (8,71)	2
**COA**	63%	8%	0	8%	45%	55%	17 (8,71)	4
**Misc**	52%	9%	7%	7%	63%	65%	81 (14,174)	15
**Total**	**57%**	**8%**	**8%**	**7%**	**46%**	**55%**	**38 (6,121)**	**144**

**Notes**:

*excludes 144 children who did not have an intervention;

† **Number (%) of children with missing data**: Sex n = 129(3%); Unstable clinical status n = 1784(46%); age at first procedure n = 50(1% of 3753 children who had an intervention); Preterm birth/Down's syndrome (DS)/Non-DS non-cardiac malformations/Additional cardiac defects: no missing data.

**Key**: **IQR** interquartile range; **preterm** <37 completed weeks gestation at birth; **Add. cardiac defects** - in addition to primary CHD diagnosis; **unstable clinical status** on first admission was defined as unstable if one or more of the following symptoms/signs were present: unwell = significant pallor, breathlessness or sweating, intubated, mechanically ventilated, hypotension = systolic blood pressure [SBP] <50 mmHg, hypertensive = SBP >100 mmHg, cardiac or respiratory arrest, metabolic acidosis, requiring adrenaline or high dose inotropic support; **HLH/MA** hypoplastic left heart and/or mitral atresia; **TA** tricuspid atresia; **DIV** double inlet ventricle; **PA+IVS** pulmonary atresia with intact ventricular septum; **PA+VSD** pulmonary atresia with ventricular septal defect; **CAT** common arterial trunk; **CAVSD** complete atrioventricular septal defect; **TGA** transposition of the great arteries; **TOF** tetralogy of Fallot; **TAPVC** total anomalous pulmonary venous connection; **VSD** ventricular septal defect; **AS** aortic stenosis; **PS** pulmonary stenosis; **COA** coarctation of the aorta; **Misc** miscellaneous cardiac defects (not included within other categories).

Hierarchical models of mortality risk were developed to take account of the grouping of individual children within cardiac centres and the correlation of procedure-related data within the individual child. For each imputed dataset multilevel discrete-time event models [19], [20] were constructed to predict mortality risk from birth to follow-up in 2007, when all surviving children were aged 12 to 15 years. The final results reported are those averaged over these datasets according to ‘Rubin's rules’. The results from these analyses using imputed datasets were compared with those using the ‘complete case’ datasets (including only those children with complete data).

Analyses were undertaken using Stata SE 11 (Timberlake Consulting) and MLwiN 2.18–2.20 [17]

## Results

Almost one-quarter of the cohort died (n = 932 deaths) and, of these, 727 (78%) deaths occurred within the first year and 323 (35%) within the first month of life. Although the risk of death was lower after the first year of life, 20% of all cohort deaths occurred after one year of age. Death occurred before intervention, or parents chose palliative care, for 144 (4%) children who died without any procedure; most (n = 63; 44%) of these children had hypoplastic left heart and/or mitral atresia (HLH/MA). The median age at death for children who died without undergoing an intervention was 8.5 days (IQR 3, 75.5 days) and only one-third survived beyond the first month of life. Of 723 children who survived to one year without having undergone a definitive corrective or palliative procedure, 578 (80%) died between age one and 15 years. There was no significant difference in the proportion of girls and boys who died without a procedure. Characteristics of the cohort children and procedure-related factors are presented in Table 5 and Table 6 respectively.

**Table 5:** Characteristics of individuals in the cohort (n = 3897).

Patient-specific factors	N (% of 3897)	Missing
		N (% of 3897)
**Sex**		129 (3%)
Boys	2147 (55%)	
Girls	1621 (42%)	
**Preterm birth**		0
Gestation <37 weeks	296 (8%)	
Gestation ≥37 weeks	3601 (92%)	
**Non-cardiac malformations**		0
Down's syndrome (DS)	293 (8%)	
Non-DS non-cardiac malformations	290 (7%)	
No non-cardiac malformations	3314 (85%)	
**Additional cardiac defects**		0
Isolated CHD	1826 (47%)	
Additional cardiac defects	1488 (53%)	
**Antenatal diagnosis of CHD**		0
Antenatal diagnosis	177 (5%)	
Postnatal diagnosis	3720 (95%)	
*** Factors related to management***		
**Clinical status on first admission**		1784 (46%)
Stable	953 (24%)	
Unstable	1160 (30%)	
**Age at first procedure** (*median [IQR]*)	38 (6, 121) days	
**Weight z-score at first procedure** (*median [IQR]*)	−1.7 (IQR −2.83, −0.58)	
**Height z-score at first procedure** (*median [IQR]*)	−0.8 (IQR −2.03, 0.42)	
**Number of procedures** (*median [IQR]*)	1 (1, 2)	

Notes:

IQR interquartile range;

Preterm birth - before 37 completed weeks of gestation;

Additional cardiac defects - children who had at least one structural cardiac defect in addition to their primary cardiac diagnosis;

Non-Down's syndrome non-cardiac malformations - a further 290 children had non-cardiac congenital malformations that were not Down's syndrome, including recognised syndromes such as Di George's (n = 61).

Clinical status on first admission was defined as unstable if one or more of the following symptoms/signs were present: unwell = significant pallor, breathlessness or sweating, intubated, mechanically ventilated, hypotension = systolic blood pressure [SBP] <50 mmHg, hypertensive = SBP >100 mmHg, cardiac or respiratory arrest, metabolic acidosis, requiring adrenaline or high dose inotropic support.

**Table 6:** Characteristics of procedure-related factors (6351 procedures in 3753 individuals).

Procedure-related factors	Details of procedures	
	N (% of 6351 procedures)	
**Pre-procedure variables:**		
Any pre-procedure complications/support	786 (12%)*	
*Intubated*	*590*	
*Inotropic support*	*252*	
*Acidosis*	*159*	
*Hypotensive (systolic BP <50 mmHg)*	*111*	
*Sepsis*	*93*	
*Hypertensive (systolic BP >100 mmHg)*	*51*	
*Seizures*	*46*	
**Intra-procedure variables:**		
Procedures not requiring bypass‡	2486 (39%)	
Cardiopulmonary bypass (CPB) time†	2346	*median = 87(IQR 60,129) minutes*
Circulatory arrest (CA) time†	1059	*median = 30 (IQR 14,49) minutes*
Aortic cross-clamp (XC) time†	1980	*median = 52 (IQR 32,75) minutes*
**Post-procedure variables:**		
Any post-procedure complications	816 (13%)*	
*Sepsis*	*356*	
*Re-intubated (after 24 hrs extubation)*	*224*	
*Renal failure*	*202*	
*Cardiac arrest*	*169*	
*Seizures*	*113*	
*ECMO (extracorporeal membrane oxygenation)*	*47*	
*DIC (disseminated intravascular coagulopathy)*	*31*	
*Stroke*	*22*	

**Notes**:

**IQR** interquartile range; BP blood pressure; hrs hours.

*Procedures at which there was more than one pre-procedure (or post-procedure) complication are only counted once in the totals so the sum of individual complications is greater.

‡ Procedures for which cardiopulmonary bypass would not be required, e.g. catheter intervention.

† Excludes procedures in which duration is recorded as 0 minutes.

**Figure 1:**
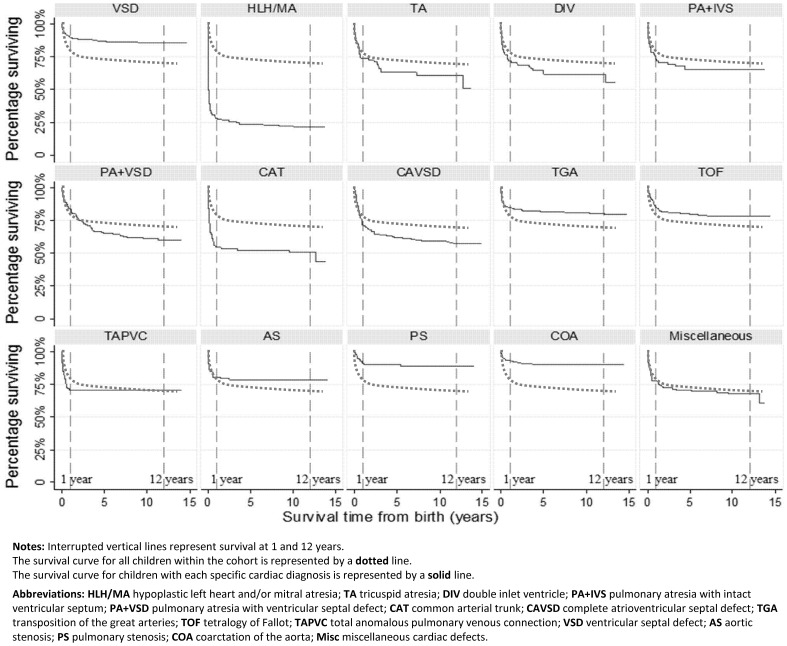
Survival from birth to 15 years by primary diagnosis (for individual diagnoses). **Notes**: Interrupted vertical lines represent survival at 1 and 12 years. The survival curve for all children within the cohort is represented by a **dotted** line. The survival curve for children with each specific cardiac diagnosis is represented by a **solid** line. **Abbreviations**: **HLH/MA** hypoplastic left heart and/or mitral atresia; **TA** tricuspid atresia; **DIV** double inlet ventricle; **PA+IVS** pulmonary atresia with intact ventricular septum; **PA+VSD** pulmonary atresia with ventricular septal defect; **CAT** common arterial trunk; **CAVSD** complete atrioventricular septal defect; **TGA** transposition of the great arteries; **TOF** tetralogy of Fallot; **TAPVC** total anomalous pulmonary venous connection; **VSD** ventricular septal defect; **AS** aortic stenosis; **PS** pulmonary stenosis; **COA** coarctation of the aorta; **Misc** miscellaneous cardiac defects.

Overall 80% (95% CI 78%–81%) of children were alive at one year and 72% [70%, 73%] at 15 years of age. Survival varied by primary cardiac diagnosis (Figure 1; Table 7). For 2,489 children remaining under follow-up after one year of age, survival between one and 15 years was 90% (95% CI 88%, 91%) overall, however for children within six diagnostic subgroups (HLH/MA, tricuspid atresia [TA], double inlet ventricle [DIV], pulmonary atresia with intact ventricular septum [PA+IVS], PA+VSD and CAVSD), survival post-infancy was lower than 90%.

**Table 7:** Data Table for Figure 1.

PRIMARY CARDIAC DIAGNOSIS	Number at birth	Survivor function	
		(95% confidence intervals)	
	*n*	At 1 year	At 12 years
Hypoplastic left heart/mitral atresia	199	**28%**(22%,35%)	**21%**(15%,27%)
Tricuspid atresia	67	**74%**(62%,83%)	**61%**(48%,72%)
Double inlet ventricle	85	**72%**(61%,80%)	**62%**(50%,71%)
Pulmonary atresia with intact ventricular septum	83	**73%**(62%,82%)	**65%**(53%,74%)
Pulmonary atresia with ventricular septal defect	151	**84%**(77%,89%)	**60%**(51%,68%)
Common arterial trunk (truncus arteriosus)	99	**55%**(44%,64%)	**51%**(40%,61%)
Complete atrioventricular septal defect	460	**71%**(67%,76%)	**57%**(52%,62%)
Transposition of the great arteries	597	**85%**(82%,88%)	**81%**(77%,84%)
Tetralogy of Fallot	361	**85%**(81%,88%)	**79%**(74%,83%)
Total anomalous pulmonary venous connection	150	**71%**(62%,78%)	**71%**(62%,78%)
Ventricular septal defect	760	**90%**(87%,92%)	**86%**(83%,88%)
Aortic stenosis	107	**81%**(72%,87%)	**78%**(69%,85%)
Pulmonary stenosis	194	**91%**(85%,94%)	**89%**(83%,93%)
Coarctation of the aorta	395	**94%**(90%,96%)	**90%**(86%,93%)
Miscellaneous	189	**77%**(70%,83%)	**68%**(60%,75%)

In univariable models involving *all* children followed from birth, preterm birth (hazard ratio [HR] 1.43 [95% CI: 1.16, 1.77]), non-Down's syndrome non-cardiac malformations (HR 1.56 [1.27, 1.93]), cardiac defects additional to the primary diagnosis (HR 1.24 [1.09, 1.41]) and unstable clinical status on first admission (HR 1.43 [1.07, 1.90]) were statistically significant predictors of higher mortality. Relative to the largest subgroup (ventricular septal defect [VSD]), all primary diagnostic subgroups except pulmonary stenosis (PS) and aortic coarctation (COA) were associated with increased mortality.

In the multivariable model including all children and adjusted for centre effects, higher mortality up to age 15 years was associated with female sex (HR 1.25 [1.06, 1.47]), preterm birth (HR 1.44 [1.15, 1.79]), non-Down's syndrome non-cardiac malformations (HR 1.49 [1.20, 1.86]), additional cardiac defects (HR 1.23 [1.07, 1.42]) and, relative to VSD, all primary diagnoses except for PS and COA (data not shown). When procedure-related risk factors were included in the multivariable model, thus excluding children who died without a procedure, independent predictors of higher mortality risk were female sex, preterm birth, additional cardiac defects, pre-procedure sepsis or hypertension, post-procedure seizures, cardiac arrest, renal failure, stroke, sepsis or disseminated intravascular coagulopathy (DIC), lower weight or height z-score, younger age at first procedure, or longer duration of cardiopulmonary bypass or cardiac arrest (Table 8). Non-Down's syndrome non-cardiac malformation and postoperative ECMO were not significant predictors of higher mortality. Mortality risk for children with TGA and COA was similar to VSD, and significantly higher than VSD for all other CHD subgroups.

**Table 8:** Multivariable survival analysis using multiple imputation (n = 3725*).

Variable	Reference Category	Category	Hazard Ratio			95% CI			P-value
						*lower*		*upper*	
**Sex**	*Boys*								
		*Girls*	1.51			1.24		1.84	<0.0001
**Birth gestation**	*Term (≥37 weeks)*								
		*Preterm (<37 weeks gestation)*	1.31			1.01		1.69	0.042
**Non-cardiac malformations**	*None*								
		*Downs Syndrome*	0.87			0.62		1.21	0.403
		*Non- Downs Syndrome*	1.26			0.97		1.64	0.085
**Additional cardiac defects**	*Isolated CHDs*								
		*Additional defects*	1.34			1.13		1.57	0.001
**Clinical status on admission**	*Stable*								
		*Unstable*	1.17			0.82		1.66	0.383
**Primary diagnoses**	*VSD*								
		*Hypoplastic left heart/mitral atresia*	7.58			5.20		11.04	<0.0001
		*Tricuspid atresia*	4.05			2.45		6.69	<0.0001
		*Double inlet ventricle*	3.31			2.07		5.29	<0.0001
		*Pulmonary atresia with intact ventricular septum*	2.98			1.75		5.08	<0.0001
		*Pulmonary atresia with ventricular septal defect*	2.98			2.01		4.42	<0.0001
		*Common arterial trunk (truncus arteriosus)*	2.53			1.65		3.89	<0.0001
		*Complete atrioventricular septal defect*	3.96			2.88		5.42	<0.0001
		*Transposition of the great arteries*	1.36			0.97		1.90	0.074
		*Tetralogy of Fallot*	2.55			1.78		3.66	<0.0001
		*Total anomalous pulmonary venous connection*	2.11			1.35		3.32	0.001
		*Aortic stenosis*	1.85			1.10		3.13	0.021
		*Pulmonary stenosis*	1.85			1.06		3.22	0.030
		*Coarctation of the aorta*	1.11			0.71		1.75	0.645
		*Miscellaneous*	2.52			1.67		3.80	<0.0001
**Procedure- related variables: constant**									
**Age at first procedure**		*per month increase*		0.90	0.86			0.93	<0.0001
**Procedure- related variables: time-varying**									
**Pre-procedure complications**	*None*								
		*Inotropic support*	1.57		0.83		2.97		0.162
		*Intubation*	0.83		0.48		1.42		0.497
		*Seizures*	1.38		0.48		4.01		0.551
		*Sepsis*	2.69		1.27		5.71		0.010
		*Metabolic acidosis*	0.93		0.43		2.04		0.859
		*Hypertension*	3.78		1.23		11.60		0.020
		*Hypotension*	1.84		0.99		3.43		0.053
**Post-procedure complications**	*None*								
		*Seizures*	1.54		1.05		2.26		0.027
		*Cardiac arrest*	4.98		3.78		6.55		<0.0001
		*Renal failure*	1.78		1.27		2.48		0.001
		*Stroke*	2.03		1.05		3.90		0.034
		*Sepsis*	1.46		1.09		1.95		0.010
		*Disseminated intravascular coagulopathy (DIC)*	6.44		3.78		10.95		<0.0001
		*Extra-corporeal membrane oxygenation (ECMO)*	1.42		0.83		2.45		0.200
		*Re-intubation (after 24 hours extubation)*	0.97		0.73		1.30		0.851
**Weight and height**									
z-score weight		*per one z-score unit increase*	0.92		0.85		1.00		0.048
z-score height		*per one z-score unit increase*	0.87		0.81		0.93		<0.0001
**Intra-procedure**									
Cardiopulmonary bypass duration		*per 10 min increase*	1.03		1.01		1.05		0.001
Cardiac arrest duration		*per 10 min increase*	1.10		1.08		1.12		<0.0001
Aortic cross-clamping duration		*per 10 min increase*	0.98		0.95		1.01		0.243

*Adjusted for specialist cardiac centre; excludes children who did not have an intervention or who died on same day as birth.

In sensitivity analyses results from the imputed models were compared with those using complete cases; the imputed data analyses provided greater precision with no significant difference in the magnitude and direction of effect. During stepwise development of the model, the variable representing definitive surgery did not significantly improve the model and was excluded. However in a sensitivity analysis involving only 606 children who had complete data and were alive at age one year, mortality risk was significantly lower for those who had experienced definitive surgery by age one year than for those who did not (HR 0.19, 95%CI 0.05, 0.73; p = 0.015).

## Discussion

In this UK-wide cohort involving 3897 children with serious CHDs, mortality was 20% in the first year of life. An additional 8% of the cohort died between one and 15 years of age, thus 20% of all deaths within the cohort occurred after the first year of life. There were 144 children in the cohort who died without any intervention during the first year of life. Overall survival was 79.8% at one year and 71.7% at 15 years of age with variation by primary cardiac diagnosis, thus for children who survived the first year survival into adulthood was generally good. Children with functional single ventricles (HLH/MA, TA and DIV) experienced the highest mortality overall but for children surviving to one year of age, those with PA+VSD and CAVSD had the worst post-infant survival rates.

Primary cardiac diagnosis was an important independent predictor of mortality risk up to 15 years; children with TGA, COA and VSD had the lowest mortality risk. Higher mortality risk regardless of intervention was independently associated with female sex, preterm birth and having a cardiac defect in addition to the primary cardiac diagnosis. For children who had at least one procedure, higher mortality risk was associated with earlier age at first procedure, pre-procedural sepsis and hypertension (systolic BP>100 mHg), post-procedural complications (including cardiac arrest, stroke, renal failure, seizures and sepsis) and increased duration of cardiopulmonary bypass or cardiac arrest.

Longitudinal cohort studies present important methodological challenges for survival analysis, including the need to model hierarchical data structures, repeated procedures, and to address missing data. An important strength of our study is the development of multilevel models that allowed us to link procedure-related factors across the child's lifecourse and to explicitly order these in time. Children in the cohort underwent varying numbers of procedures, which did not occur at fixed ages, and earlier postoperative experience could influence later management. Despite recognition of the complexity of survival analysis in children with CHDs, for whom both predictors and mortality are time-dependent [11], examples of survival models including time-varying covariates are rare and few previous studies have explicitly stated the temporal ordering of childhood factors and their inter-relationships, directly or through mediating factors, with mortality. We addressed the methodological challenges of repeat observations, and also adjusted for the effects of clustering by cardiac centre, through the development of discrete-time hierarchical survival models [19], [20] and multilevel multiple imputation [44], [45] of missing values. The use of imputed datasets, by allowing us to include all cohort children and procedures in the analyses, reduced the likelihood of bias that can result from restricting analyses to the small proportion of children with complete data [21]. Our sensitivity analyses evaluating the effect of multiple imputation in comparison with complete-case datasets, demonstrated that imputed data contributed to greater precision in mortality estimates and ensured that the impact of less commonly reported factors, such as pre- and post-procedural complications, was quantifiable.

Although we were prevented by our governance permissions [22] from accessing routine mortality data to check for deaths amongst children who clinicians presumed to be surviving, all reported deaths were confirmed against public death registrations. As children with serious CHDs remain under review throughout life, we believe that our ascertainment of deaths through cardiologists, hospital records and GPs was complete.

The heterogeneity of CHDs also presents a significant problem for population-based analyses [23], [24] and variability in classification systems often limits comparisons between studies. Our simple hierarchical classification adapted from Wren [14] and validated on data available from medical case notes, ensured that each child was assigned to only one CHD subgroup and avoided double-counting of children with multiple cardiac defects. As similar classifications have been successfully applied in other UK studies [9], [25] [26], this provides a sound basis for cross-study comparisons.

Patients do not pay for care within the UK healthcare system, thus there were no financial barrier to accessing surgery and deaths in children who were offered only palliative medical care were related to the inoperability of the defect. As with all long-term follow-up, early management of cohort members who were born in 1992–1995 was determined by clinical era. Although our cohort has its inception after the introduction of neonatal cardiac surgery and key procedures, such as the arterial switch operation, paediatric cardiac surgical and intensive care technologies have continued to advance, notably for children with HLH for whom Norwood-type surgery is now widely available, or with TOF for whom neonatal surgery is now common. Nonetheless, surgical management has not altered markedly for many CHD subgroups. Advances in fetal screening s well as surgery may also alter the proportion of children who would be offered palliative medical care in current practice compared our cohort. Whereas fetal screening detected around 23% of severe CHDs in 1993–94 [13], this has now increased to detection of around one-third of cases [27], which may mean that more children born today would commence specialist care at birth than in our cohort. The findings from our cohort should therefore be interpreted with caution within specific diagnostic subgroups where surgical techniques or screening detection have altered significantly as there may be improved survival for children born today. Nevertheless we believe our results are still likely to remain relevant overall to children born and operated with CHDs today, particularly for diagnostic subgroups in which significant improvements in early postoperative survival have not been seen.

There are relatively few population-based observational cohort studies that describe mortality and survival up to age 12–15 years for children affected by CHDs (Table 9). Although we also present some findings from our model including children who remained unoperated at the time of death, the majority of these children died in the first month after birth, many had HLH and palliative care was chosen by some parents; management of such severe and complex cases has continued to advance in the last decade. Thus our main model excludes these unoperated cases and focuses on survival from birth for the majority of children in the cohort, who did undergo an intervention.

**Table 9:** Population-based cohort studies reporting mid- and long-term survival for children with congenital heart defects.

Lead author, publication year	Region/State, Country	Period	Study population (n)	Method/Design; Outcome measure	Period of follow-up	Survival/mortality
**Morris, 1991 [29]**	Oregon, US	Operated 1958–1989	Children aged <18 years (n = 2,701)	Retrospective review of paediatric cardiac procedures (for 8 CHD types); Death after surgery	Up to 25 years after surgery	Varying by CHD: 64% survival at 15 years after surgery (transposition) to 98% survival (atrial septal defect)
**Samanek, 1992 [28]**	Bohemia, Czech Republic	Diagnosed 1952–1979	Deaths aged ≤15 years (n = 946)	Retrospective review of death registrations; Death ≤15 years old	Up to 16 years of age	71% survival at 1 year of age; 67% survival at 15 years of age
**Meberg, 2000 [31]**	Vestfold County, Norway	Diagnosed 1982–1996	Children diagnosed clinically or at post-mortem (n = 360)	Prospective follow-up after diagnosis; Death ≤18 years old	Mean 9.5 years (range 3–18 years)	Overall 12% mortality at end of follow-up
**Nieminen, 2001 [10]**	Finland	Operated 1953–1989	Children aged <15 years old at surgery (n = 6,461)	Retrospective review of paediatric cardiac procedures (all CHDs); Death after surgery	Mean 22 years after surgery (range 9–45 years)	Overall 7% surgical mortality; 78% survival at 45 years after surgery
**Wren, 2001 [9]**	Northern Region, UK	Born 1985–1994	Children diagnosed clinically or at post-mortem (n = 1,942)	Prospective follow-up from birth/diagnosis; Death ≤15 years old	Up to 16 years of age	82% survival at 1 year of age; *Predicted 78% survival to 16 years of age*
**Moons, 2009 [32]**	Belgium	Born 2002	Children diagnosed clinically (n = 921)	Prospective follow-up from birth/diagnosis; Death ≤5 years old	Up to 5 years of age	96% survival at 5 years of age
**Larsen, 2011 [30]**	Western Region, Denmark	Operated 1996–2002	Children operated for CHD (n = 801)	Prospective follow-up after cardiac surgery; Death after surgery	Median 8.2 years after surgery (range 6–12 years)	86% survival at 8.2 years after surgery

Reviewing all child CHD deaths in Bohemia between 1952 and 1979, Samanek reported that ‘natural’ survival before the widespread introduction of paediatric cardiac surgery was 67% at 15 years [28]. Three further studies investigated survival in large surgical cohorts [10], [29], [30], including a Finnish population-based study between 1953 and 1989 in which survival was reported as 78% at 45 years after surgery, significantly lower than for the unaffected population. Only three studies have reported survival after diagnosis in early life, including children who remained unoperated as well as those undergoing surgery, of which two present directly observed survival from diagnosis [31] [32]. These two studies, representing contemporary management, documented better overall survival (Table 10) than we observed but up to 40% of children survived without any interventions suggesting that, in comparison with the UKCSCHD, they included a higher proportion of mild CHDs that were compatible with survival without intervention.

**Table 10:** Childhood survival by primary cardiac diagnosis reported within population-based cohort studies published since 1990.

PRIMARY CARDIAC DIAGNOSIS	UKCSCHD	Samanek, 1992 [28]	Meberg, 2000 [31]	Wren, 2001 [9]
	At age 12 years (‘observed’)	At age 15 years (‘natural’)*	At age 9.5 years (‘observed’)	At age 15 years (‘predicted’)§
Hypoplastic left heart	**21%**	**11%**	**22%**	**0**
Pulmonary atresia	**60–65%**	**30%**	**75%**	**40%**
Common arterial trunk (truncus arteriosus)	**51%**	**11%**	**50%**	**31%**
Complete atrioventricular septal defect	**57%**	**49%**	**55%**	**55%**
Transposition of the great arteries	**81%**	**38%**	**85%**	**67%**
Tetralogy of Fallot	**79%**	**86%**	**82%**	**84%**
Total anomalous pulmonary venous connection	**71%**	**0**	**-**	**70%**
Ventricular septal defect	**86%**	**76%**	**97%**	**91%**
Aortic stenosis	**78%**	**84%**	**92%**	**66%**
Pulmonary stenosis	**89%**	**94%**	**93%**	**92%**
Coarctation of the aorta	**90%**	**68%**	**84%**	**85%**

**Notes** :

This table compares defect-specific survival reported in childhood within four population-based cohorts that include all children diagnosed in infancy even if no intervention was performed. Most studies present ‘observed’ cohort survival from diagnosis or first surgery during infancy, with the exception of:

*Samanek presents ‘natural’ survival in an era prior to widespread surgical correction.

§ Wren presents ‘predicted’ survival up to age 15 years based on observed survival to age 1 year and survival from 1 to 15 years estimated from a review of the published literature.

Surgical case series have highlighted important associations between increased postoperative mortality risk and factors such as low birth weight [33], [34], preterm birth [35], sudden clinical deterioration in the neonatal period [36], [37], and procedure-associated complications, including sepsis and renal failure [38]. Several studies have demonstrated the detrimental impact of longer intra-procedure duration of cardiopulmonary bypass and cardiac arrest [39]–[42], also observed in our cohort, although most previous authors estimated the effect at a single procedure only rather than repeated exposure over multiple procedures. Moreover Kang has cautioned that longer duration of cardiopulmonary bypass may only reflect technically more difficult surgery [41]

Although our findings indicate that girls with CHDs are at significantly higher risk of death, published evidence for sex differences in mortality for individuals with CHDs remains inconclusive. Morris [29] demonstrated that girls with COA and TGA had higher mortality prior to intervention, whereas Fyler [43] reported a higher death rate for boys during the first year of life. Other authors have highlighted that girls experience higher perioperative mortality related to paediatric cardiac surgery [44]–[47]. As the ratio of boys to girls affected varies by specific cardiac defect, these sex differences may be confounded by the severity of cardiac diagnosis. Nevertheless children's cardiac size and function, lung development and immune responses have been shown to vary by sex [47]–[51], therefore biological differences also provide a plausible explanation for sex differences in mortality risk and would merit further research.

In accordance with previous reports [52], [53], we found no independent influence on mortality associated with Down's syndrome. Eskedal [52] has highlighted that children with CHDs who have non-cardiac malformations other than Down's syndrome experience higher mortality than those with isolated cardiac defects. In our cohort, non-Down's non-cardiac malformations were significant predictors of mortality when children who died without intervention were included in the analysis, suggesting that these malformations do contribute to mortality in this subgroup.

It is now over a decade since the Bristol Inquiry addressed concerns about the care of children receiving cardiac surgery at the Bristol Royal Infirmary [54]. Expert evidence presented to the Inquiry emphasised the lack of data on long-term outcomes relevant to children and their families [55]. The Central Cardiac Audit Database (CCAD) now routinely collects national outcome data for cardiac procedures, including survival up to one year after paediatric cardiac intervention; these data are published through the National Institute for Cardiac Outcomes Research (NICOR) Congenital Heart Disease Portal [56]

While routine audit remains essential to monitoring outcomes for children receiving interventions for CHDs in the UK [57], a procedure-based system excludes children who never receive an intervention and often fails to capture late mortality and broader health outcomes, such as educational achievement or quality of life. Improved record linkage between multiple routinely collected data sources, supplemented by focused observational or clinical studies, could enrich current routine data collection and provide more efficient use of existing resources for extended follow-up.

## Conclusions

The UKCSCHD has provided a unique opportunity to observe survival and wider health outcomes throughout childhood in a prospectively ascertained UK-wide cohort that is largely representative of contemporary management. We found that infant mortality under age one year for children with serious CHDs was over 30 times greater than for the general population, estimated at six per 1,000 live births [58]. It is also clear from our data that whilst children with CHDs were still most likely to die in infancy in this cohort, over 20% of CHD-related deaths in childhood took place after the first year of life. Surgical management of serious CHDs carries an initial perioperative risk of death or neurological injury in childhood, but the altered physiology of the operated heart may also result in reduced ability to cope with ageing or cardiac stressors arising in adult life. Long-term survival and health outcomes are influenced by health events across the lifecourse thus optimising the health experience of preschool and school-age children with CHDs is as relevant to future improvements in survival and quality of life of adults with CHDs as successful early surgical repair. Crucially cardiac diagnosis remained an important predictor of outcome particularly as this influenced the type of definitive procedure, whether this was palliative or corrective, and the age at which it was performed. However, we also identified procedure-related predictors of mortality that may be amenable to modification in order to effect further advances in survival or improve health outcomes after surgery. Investigation into the biological mechanisms contributing to higher mortality for girls is warranted to explore the potential for improving future outcomes and reducing health inequalities.

## References

[1] Dadvand P, Rankin J, Shirley MD, Rushton S, Pless-Mulloli T (2009) Descriptive epidemiology of congenital heart disease in Northern England. Paediatr Perinat Epidemiol 23: 58–65. 19228315 10.1111/j.1365-3016.2008.00987.x

[2] Lee K, Khoshnood B, Chen L, Wall SN, Cromie WJ, et al (2001) Infant mortality from congenital malformations in the United States, 1970–1997. Obstet Gynecol 98: 620–627. 11576578 10.1016/s0029-7844(01)01507-1

[3] Khoshnood B, De Vigan C, Vodovar V, Goujard J, Lhomme A, et al (2005) Trends in prenatal diagnosis, pregnancy termination, and perinatal mortality of newborns with congenital heart disease in France, 1983–2000: a population-based evaluation. Pediatrics 115: 95–101. 15629987 10.1542/peds.2004-0516

[4] Samanek M, Voriskova M (1999) Congenital heart disease among 815,569 children born between 1980 and 1990 and their 15-year survival: a prospective Bohemia survival study. Pediatr Cardiol 20: 411–417. 10556387 10.1007/s002469900502

[5] Macmahon B, McKeown T, Record RG (1953) The incidence and life expectation of children with congenital heart disease. Br Heart J 15: 121–129. 13041990 10.1136/hrt.15.2.121 PMC479477

[6] Dastgiri S, Gilmour WH, Stone DH (2003) Survival of children born with congenital anomalies. Arch Dis Child 88: 391–394. 12716706 10.1136/adc.88.5.391 PMC1719557

[7] Freedom RM, Lock J, Bricker JT (2000) Pediatric cardiology and cardiovascular surgery: 1950–2000. Circulation 102 20 Suppl 4 IV58–68. 11080133 10.1161/01.cir.102.suppl_4.iv-58

[8] NHS Information Centre (2009) National Audit of Congenital Heart Disease: Executive Summary 2009. Leeds, UK: NHS Information Centre. Available: http://www.hscic.gov.uk/catalogue/PUB02661/nati-cong-hear-dise-exec-summ-audi-2009-rep.pdf Accessed: 17 July 2014.

[9] Wren C, O'Sullivan JJ (2001) Survival with congenital heart disease and need for follow up in adult life. Heart 85: 438–443. 11250973 10.1136/heart.85.4.438 PMC1729699

[10] Nieminen HP, Jokinen EV, Sairanen HI (2001) Late results of pediatric cardiac surgery in Finland: a population-based study with 96% follow-up. Circulation 104: 570–575. 11479255 10.1161/hc3101.093968

[11] McCrindle BW (2001) Considerations in the appraisal of mortality associated with congenital cardiac lesions. Semin Thorac Cardiovasc Surg Pediatr Card Surg Annu 4: 244–255. 11460988

[12] Ben-Shlomo Y, Kuh D (2002) A life course approach to chronic disease epidemiology: conceptual models, empirical challenges and interdisciplinary perspectives. Int J Epidemiol 31: 285–293. 11980781

[13] Bull C (1999) Current and potential impact of fetal diagnosis on prevalence and spectrum of serious congenital heart disease at term in the UK. British Paediatric Cardiac Association. Lancet 354: 1242–1247. 10520632 10.1016/s0140-6736(99)01167-8

[14] Wren C, Richmond S, Donaldson L (2000) Temporal variability in birth prevalence of cardiovascular malformations. Heart 83: 414–419. 10722540 10.1136/heart.83.4.414 PMC1729354

[15] Lane DA, Lip GY, Millane TA (2002) Quality of life in adults with congenital heart disease. Heart 88: 71–75. 12067950 10.1136/heart.88.1.71 PMC1767157

[16] Cole TJ, Freeman JV, Preece MA (1998) British 1990 growth reference centiles for weight, height, body mass index and head circumference fitted by maximum penalized likelihood. Stat Med 17: 407–429. 9496720

[17] Rasbash J, Charlton C, Browne WJ, Healy M, Cameron B (2009) MLwiN version 2.1. University of Bristol, UK: Centre for Multilevel Modelling.

[18] Goldstein H (2009) REALCOM-Impute: Multiple Imputation using MLwiN, User Guide. University of Bristol: Centre for Multilevel Modelling.

[19] Goldstein H, Browne W, Rasbash J (2002) Multilevel modelling of medical data. Stat Med 21: 3291–3315. 12375305 10.1002/sim.1264

[20] Goldstein H (2011) Multilevel Statistical Models. Chichester, UK: John Wiley and Sons.

[21] Kenward MG, Carpenter J (2007) Multiple imputation: current perspectives. Stat Methods Med Res 16: 199–218. 17621468 10.1177/0962280206075304

[22] Knowles RL, Bull C, Wren C, Dezateux C (2011) Ethics, governance and consent in the UK: implications for research into the longer-term outcomes of congenital heart defects. Arch Dis Child 96: 14–20. 19773220 10.1136/adc.2008.152975

[23] Brown KL, Crowe S, Pagel C, Bull C, Muthialu N, et al (2013) Use of diagnostic information submitted to the United Kingdom Central Cardiac Audit Database: development of categorisation and allocation algorithms. Cardiol Young 23: 491–498. 23025920 10.1017/S1047951112001369

[24] Riehle-Colarusso T, Strickland MJ, Reller MD, Mahle WT, Botto LD, et al (2007) Improving the quality of surveillance data on congenital heart defects in the metropolitan Atlanta congenital defects program. Birth Defects Res A Clin Mol Teratol 79: 743–753. 17990334 10.1002/bdra.20412

[25] Billett J, Majeed A, Gatzoulis M, Cowie M (2008) Trends in hospital admissions, in-hospital case fatality and population mortality from congenital heart disease in England, 1994 to 2004. Heart 94: 342–348. 17646196 10.1136/hrt.2006.113787

[26] Crowe S, Brown KL, Pagel C, Muthialu N, Cunningham D, et al (2013) Development of a diagnosis- and procedure-based risk model for 30-day outcome after pediatric cardiac surgery. J Thorac Cardiovasc Surg 145: 1270–1278. 22818122 10.1016/j.jtcvs.2012.06.023

[27] Wren C, Reinhardt Z, Khawaja K (2008) Twenty-year trends in diagnosis of life-threatening neonatal cardiovascular malformations. Arch Dis Child Fetal Neonatal Ed 93: F33–35. 17556383 10.1136/adc.2007.119032

[28] Samanek M (1992) Children with congenital heart disease: probability of natural survival. Pediatr Cardiol 13: 152–158. 1603715 10.1007/BF00793947

[29] Morris CD, Menashe VD (1991) 25-year mortality after surgical repair of congenital heart defect in childhood. A population-based cohort study. JAMA 266: 3447–3452. 1744959

[30] Larsen SH, Emmertsen K, Johnsen SP, Pedersen J, Hjortholm K, et al (2011) Survival and morbidity following congenital heart surgery in a population-based cohort of children–up to 12 years of follow-up. Congenit Heart Dis 6: 322–329. 21418533 10.1111/j.1747-0803.2011.00495.x

[31] Meberg A, Otterstad JE, Froland G, Lindberg H, Sorland SJ (2000) Outcome of congenital heart defects–a population-based study. Acta Paediatr 89: 1344–1351. 11106048 10.1080/080352500300002552

[32] Moons P, Sluysmans T, De WD, Massin M, Suys B, et al (2009) Congenital heart disease in 111 225 births in Belgium: birth prevalence, treatment and survival in the 21st century. Acta Paediatr 98: 472–477. 19046347 10.1111/j.1651-2227.2008.01152.x

[33] Oppido G, Napoleone CP, Formigari R, Gabbieri D, Pacini D, et al (2004) Outcome of cardiac surgery in low birth weight and premature infants. Eur J Cardiothorac Surg 26: 44–53. 15200978 10.1016/j.ejcts.2004.04.004

[34] Padley JR, Cole AD, Pye VE, Chard RB, Nicholson IA, et al (2011) Five-year analysis of operative mortality and neonatal outcomes in congenital heart disease. Heart Lung Circ 20: 460–467. 21514216 10.1016/j.hlc.2011.03.009

[35] Tanner K, Sabrine N, Wren C (2005) Cardiovascular malformations among preterm infants. Pediatrics 116: e833–e838. 16322141 10.1542/peds.2005-0397

[36] Brown KL, Ridout DA, Hoskote A, Verhulst L, Ricci M, et al (2006) Delayed diagnosis of congenital heart disease worsens pre-operative condition and outcome of surgery in neonates. Heart 92: 1298–1302. 16449514 10.1136/hrt.2005.078097 PMC1861169

[37] Bonnet D, Coltri A, Butera G, Fermont L, Le Bidois J, et al (1999) Detection of transposition of the great arteries in fetuses reduces neonatal morbidity and mortality. Circulation 99: 916–918. 10027815 10.1161/01.cir.99.7.916

[38] Brown KL, Ridout DA, Goldman AP, Hoskote A, Penny DJ (2003) Risk factors for long intensive care unit stay after cardiopulmonary bypass in children. Crit Care Med 31: 28–33. 12544989 10.1097/00003246-200301000-00004

[39] Vogt PR, Carrel T, Pasic M, Arbenz U, von Segesser LK, et al (1994) Early and late results after correction for double-outlet right ventricle: uni- and multivariate analysis of risk factors. Eur J Cardiothorac Surg 8: 301–307. 8086177 10.1016/s1010-7940(05)80090-9

[40] Nollert G, Fischlein T, Bouterwek S, Bohmer C, Klinner W, et al (1997) Long-term survival in patients with repair of tetralogy of Fallot: 36- year follow-up of 490 survivors of the first year after surgical repair. J Am Coll Cardiol 30: 1374–1383. 9350942 10.1016/s0735-1097(97)00318-5

[41] Kang N, Cole T, Tsang V, Elliott M, de Leval M (2004) Risk stratification in paediatric open-heart surgery. Eur J Cardiothorac Surg 26: 3–11. 15200974 10.1016/j.ejcts.2004.03.038

[42] Greeley WJ, Kern FH, Ungerleider RM, Boyd JL 3rd, Quill T, et al (1991) The effect of hypothermic cardiopulmonary bypass and total circulatory arrest on cerebral metabolism in neonates, infants, and children. J Thorac Cardiovasc Surg 101: 783–794. 2023435

[43] Fyler DC (1980) Report of the New England Regional Infant Cardiac Program. Pediatrics 65: 375–461. 7355042

[44] Chang RK, Chen AY, Klitzner TS (2002) Female sex as a risk factor for in-hospital mortality among children undergoing cardiac surgery. Circulation 106: 1514–1522. 12234958 10.1161/01.cir.0000029104.94858.6f

[45] Klitzner TS, Lee M, Rodriguez S, Chang RK (2006) Sex-related disparity in surgical mortality among pediatric patients. Congenit Heart Dis 1: 77–88. 18377550 10.1111/j.1747-0803.2006.00013.x

[46] Seifert HA, Howard DL, Silber JH, Jobes DR (2007) Female gender increases the risk of death during hospitalization for pediatric cardiac surgery. J Thorac Cardiovasc Surg 133: 668–675. 17320563 10.1016/j.jtcvs.2006.11.014

[47] Kochilas LK, Vinocur JM, Menk JS (2014) Age-Dependent Sex Effects on Outcomes After Pediatric Cardiac Surgery. J Am Heart Assoc 3: e000608. 24496232 10.1161/JAHA.113.000608 PMC3959673

[48] Sarikouch S, Boethig D, Beerbaum P (2013) Gender-specific algorithms recommended for patients with congenital heart defects: review of the literature. Thorac Cardiovasc Surg 61: 79–84. 23307279 10.1055/s-0032-1326774

[49] Dezateux C, Stocks J (1997) Lung development and early origins of childhood respiratory illness. Br Med Bull 53: 40–57. 9158283 10.1093/oxfordjournals.bmb.a011605

[50] Postma DS (2007) Gender differences in asthma development and progression. Gend Med 4 Suppl B: S133–146. 10.1016/s1550-8579(07)80054-4 18156099

[51] Falagas ME, Mourtzoukou EG, Vardakas KZ (2007) Sex differences in the incidence and severity of respiratory tract infections. Respir Med 101: 1845–1863. 17544265 10.1016/j.rmed.2007.04.011

[52] Eskedal L, Hagemo P, Eskild A, Aamodt G, Seiler KS, et al (2004) A population-based study of extra-cardiac anomalies in children with congenital cardiac malformations. Cardiol Young 14: 600–607. 15679995 10.1017/S1047951104006043

[53] Frid C, Bjorkhem G, Jonzon A, Sunnegardh J, Anneren G, et al (2004) Long-term survival in children with atrioventricular septal defect and common atrioventricular valvar orifice in Sweden. Cardiol Young 14: 24–31. 10.1017/s1047951104001052 15237667

[54] Bristol Royal Infirmary Inquiry (2001) Learning from Bristol: the report of the public inquiry into children's heart surgery at the Bristol Royal Infirmary 1984 -1995. Norwich, UK: The Stationery Office Ltd.

[55] Bull C (2001) Key Issues in Retrospective Evaluation of Morbidity Outcomes Following Paediatric Cardiac Surgery. Final Report of the Bristol Royal Infirmary Inquiry (CM 5207). Norwich, UK: The Stationery Office Ltd.

[56] NICOR (2012) National Institute for Cardiovascular Outcomes Research: Congenital Heart Disease Website. London, UK: University College London. Available: https://nicor4.nicor.org.uk/CHD/an_paeds.nsf/vwContent/home?Opendocument Accessed: 17 July 2014.

[57] Gibbs JL, Monro JL, Cunningham D, Rickards A (2004) Survival after surgery or therapeutic catheterisation for congenital heart disease in children in the United Kingdom: analysis of the central cardiac audit database for 2000-1. BMJ 328: 611. 14982866 10.1136/bmj.38027.613403.F6 PMC381132

[58] Office for National Statistics (2010) Infant and perinatal mortality by health areas in England and Wales, 2009. Office for National Statistics Statistical Bulletin. Newport, UK: Office for National Statistics.

